# Immunomodulatory and anti-inflammatory effects of hydro-ethanolic extract of *Ocimum basilicum* leaves and its effect on lung pathological changes in an ovalbumin-induced rat model of asthma

**DOI:** 10.1186/s12906-019-2765-4

**Published:** 2019-12-04

**Authors:** Naima Eftekhar, Ali Moghimi, Nema Mohammadian Roshan, Saeideh Saadat, Mohammad Hossein Boskabady

**Affiliations:** 1 Department of Biology, Faculty of Science, Ferdowsi University of Mashhad, Mashhad, Iran; 2 Department of Pathology, Faculty of Medicine, Mashhad University of Medical Sciences, Mashhad, Iran; 3 Department of Physiology, School of Medicine, Zahedan University of Medical Sciences, Zahedan, Iran; 4 Neurogenic Inflammation Research Center, Mashhad University of Medical Sciences, Mashhad, Iran; 5 Department of Physiology, Faculty of Medicine, Mashhad University of Medical Sciences, Mashhad, Iran

**Keywords:** *Ocimum basilicum*, immunological mediators, Inflammatory mediators, Asthma

## Abstract

**Background:**

Ocimum species (Lamiaceae) has been traditionally used for treatment of upper respiratory tract infections, bronchitis, coughs, sore throat, and wound healing. The Immunomodulatory and anti-inflammatory effects of hydro-ethanolic extract of *Ocimum basilicum* (*O. basilicum*) leaves was examined in ovalbumin sensitized animals.

**Methods:**

Wistar rats were divided to six groups; non-sensitized, sensitized to ovalbumin, sensitized and treated with dexamethasone (1.25 μg/mL), and *O. basilicum* extract (0.75, 1.50 and 3.00 mg/mL) in drinking water for 21 days. The levels of interleukin 4 (IL-4), interferon gamma (IFN-γ), IFN-γ/IL-4 ratio, immunoglobulin E (IgE), phospholipase A_2_ (PLA_2_) and total protein (TP) in BALF, and lung pathological changes were examined.

**Results:**

A significant increase in IL-4, IgE, PLA_2_ and TP levels, all lung pathological indices as well as significant decrease in IFN-γ/IL-4 ratio was seen in the asthmatic compared to the control rats (*P* < 0.05 to *P* < 0.001). Treatment with *O. basilicum* extract resulted in decreased IL-4, IgE, PLA_2_ and TP levels, but increased IFN-γ/IL-4 ratio compared to untreated sensitized rats (*P* < 0.01 to *P* < 0.001). The plant significantly improved the pathological changes of sensitized rats (*P* < 0.05 to *P* < 0.01). The improvement effects of higher concentrations of the *O. basilicum* extract were significantly more than those of dexamethasone (*P* < 0.05 to *P* < 0.001).

**Conclusion:**

The improvement effects of *O. basilicum* on pathological changes, immunological and inflammatory markers in sensitized rats comparable or even more potent than dexamethasone suggests the therapeutic potential of the plant in asthma.

## Background

Airway inflammation and tissue remodeling of the airway structure are two main pathophysiologic characteristic of asthma which mediated by numerous cells and their mediators [1, 2]. In addition, immune pathways driven by T-helper 2 (Th_2_) cells which produce IL-4, IL-5, and IL-13 are contributing in the pathophysiology of this disease. The imbalance of Th_1_/Th_2_ imbalance toward increased Th_2_ activity was indicated in the pathogenesis of asthma and allergies [3, 4]. Cytokines released from Th_2_ cells like IL- 4 and IL- 5 also stimulate IgE production by B cells which plays a role in allergic asthma [5]. Secretory phospholipases A_2_ (sPLA_2_) also may induce airway inflammation in asthma by release of arachidonic acid, generation of lysophospholipids, release of cytokines and the effect on inflammatory and immunological cells. In occupational asthma, increased serum total protein level was reported [6–8]. Airway remodeling which refers to the structural changes in the airways is another feature of asthma. Airway remodeling is believed to contribute to irreversible airflow obstruction and airway hyper responsiveness [9].

Currently used asthma medications reduce airway inflammation and diminish bronchospasm, but when treatment is discontinued, symptoms re-appear again [10]. Asthma has long been treated using medicinal plants in the Middle East, China and Europe [11]. Due to the derivation of many pharmaceutical drugs from plants origin, medicinal plants are seems to be the base of modern medicine [12]. Among the known medicinal herbs, the plants of genus Ocimum species, from Lamiaceae family, showed various therapeutic potentials. The leaves of these plants are used to relieve toothaches, sore throats, coughs, colds, bronchitis, laryngitis, nasal congestion and inflammation of the mouth and throat [13, 14]. Between 50 and 150 species of herbs and shrubs were found for Ocimum genus in the tropical regions of Central and South America, Africa and Asia [15]. *Ocimum basilicum* (*O. basilicum*) is one of the main species of genus Ocimum [16]. *O. basilicum* or sweet basil is an annual species, original from Asia, cultivated in all the Mediterranean and tropical countries. Traditionally *O. basilicum* has been used to treat coughs, headaches, diarrhea, constipation, warts, worms, and kidney malfunctions [15]. Iranian *O. basilicum* are used to treat throat congestions, fevers and stomachache [15]. It was shown that *O. basilicum* crude methanolic extract down regulated TNF-α, IL-1β and IL-2, and suppressed the induction of inducible nitric oxide synthase (iNOS) and the subsequent production of nitric oxide (NO) in LPS-stimulated RAW 264.7 macrophages in a time-dependent manner [17, 18]. Fixed oil of *O. basilicum* also blocked both cyclooxygenase and lipoxygenase pathways of arachidonic acid metabolism [17, 18]. The aqueous extract of *O. basilicum* and several other medicinal plants inhibited giant cell formation in co-culture of Molt-4 cells with and without HIV-1 infection and showed inhibitory activity against HIV-1 reverse transcriptase [19]. Other pharmacological effects of this plant such as anti-aging, anti-cancer [20] and immune modulatory [21] have been previously reported. Therefore, the anti-inflammatory and immune modulatory effects of this plant may result in improved asthma. In addition, the safety of the plant in animal and human models has been confirmed [22].

With regard to the different traditional and pharmacological effects of the plant, the effect of *O. basilicum* leaves extract on immunological and inflammatory factors in bronchoalveolar lavage fluid (BALF) and lung pathological changes in ovalbumin sensitized animals was evaluated in this study.

## Methods

### Preparation of the plant extract

The plant (*O. basilicum*) were collected from Mashhad area, (Razavi Khorasan province, Iran) and was identified by Mr. Joharchi in Research Center for Plant Sciences (specimen No. 12937061), the herbarium of School of Agriculture, Ferdowsi University of Mashhad as described previously [23].

To prepare macerated hydro-ethanolic extract, 100 g dried and grinded leaves of *O. basilicum* was dissolved in 1000 mL ethanol 70% in laboratory condition for 72 h. Using rotary evaporator, the solvent was removed and the yield of obtain dry extract was 19%.

### Animals and experimental groups

Forty eight male Wistar rats (weighing 200 ± 20 g, randomly divided) were purchased from Animal house, Faculty of Medicine, Mashhad University of Medical Sciences, Mashhad, Iran, and were maintained in a stainless steel cage with clean filtered air (Maximiser, Thorens Caging System Inc., Hazleton, PA, U.S.A.) with free water and food ad libitum, temperature of 22 ± 2 °C on a 12 h light/dark cycle during experimental period [24]. The ethics committee of Mashhad University of Medical Sciences approved the Animal Experiments of the study (allowance number: 930842). The studies groups were described in Table 1. The study was cried out according the regulations of the Institute of Laboratory Animals Resources Commission on Life Sciences [27].

**Table 1:** Studied groups (randomly divided)

Groups	Name	Concentration	Dose	Reference
Control	Non-sensitized rats (C)			
Sensitized	Non-treated, OA sensitized rats (S)			
	*O. basilicum* extract treatment S + OB 0.75 mg/ml S + OB 1.50 mg/ml S + OB 3.00 mg/ml	0.75 mg/ml 1.50 mg/ml 3.00 mg/ml	150 mg/kg/day 300 mg/kg/day 600 mg/kg/day	[23, 25, 26]
	Dexamethasone treatment	1.25 μg/ml	250 mg/kg/day	[24]

Addition of drugs did not change the water volume consumed by animals, which averaged 40 ml/day/rat. Accordingly, daily dose of extract and dexamethasone are as shown in the Table. (*n* = 8 in each group). The doses of *O. basilicum* and dexamethasone were chosen according the previous studies as indicated in the Table

### Animal sensitization

**Fig. 1:**
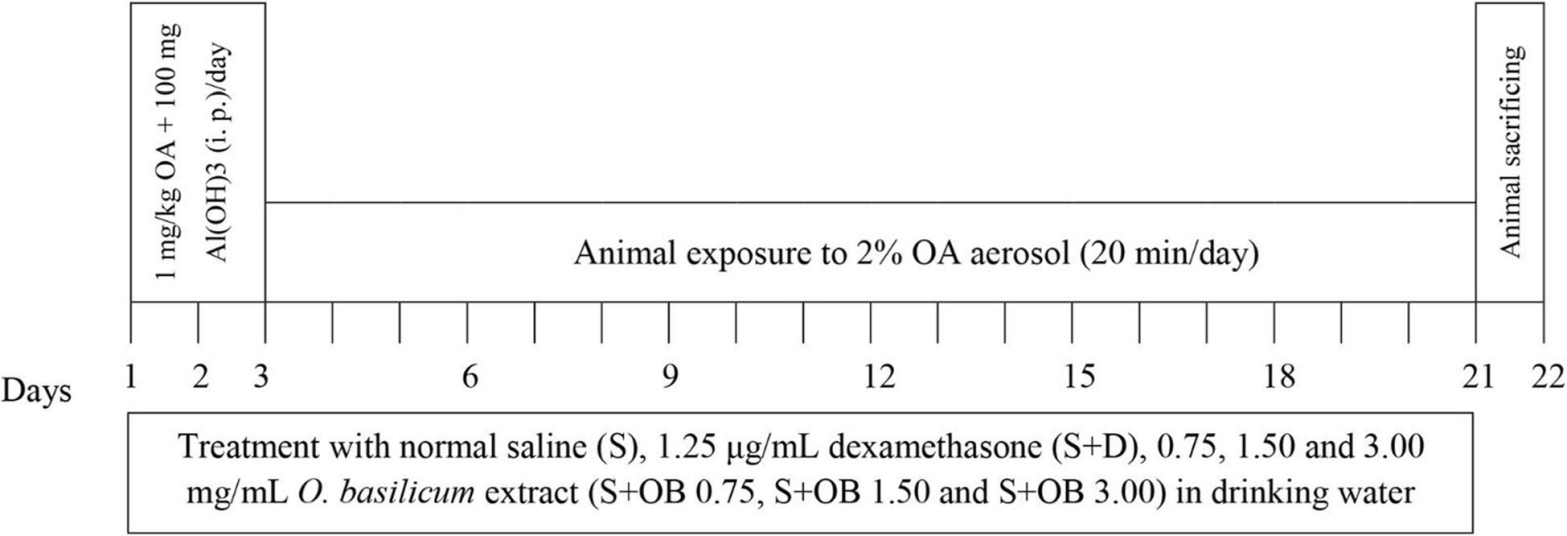
Sensitization method of rats by ovalbumin (OA), 1 mg/kg + 100 mg Al(OH)3 as adjuvant and treatment of animals

Rats were sensitized as previously described [24] and Fig. 1. Briefly 1 mg/kg ovalbumin (OA), (≥98%, CAS Number: 9006-59-1, Sigma Chemical Ltd., UK) plus 100 mg Al(OH)_3_ (CAS Number: 21645–51-2, Sigma Chemical Ltd., UK) was administered intraperitoneal (i.p.) and rats were exposed to 2% OA aerosol with air flow of 8 lit/min for 20 min/day in a 0.8 m^3^ chamber, with animal normal-breathing. Saline was used instead of the ovalbumin solution in the control rats [24].

### Immunologic and inflammatory markers measurement

One day after the end of sensitization, animals were sacrificed by i.p. administration of 50 mg/kg ketamine (CAS Number: 1867-66-9, Sigma Chemical Ltd., UK) and 5 mg/kg xylazine (CAS Number: 23076–35-9, Sigma Chemical Ltd., UK). The left lung was washed with one mL saline five times (5 mL totally). BALF was centrifuged at 2500 g at 4 °C for 10 min and supernatant was stored at − 70 °C [28] until analysis.

Cytokine and inflammatory levels including interleukin 4 (IL-4, Cat Number: ab100770, Abcam Company, Cambridge, MA, USA), interferon gamma (IFN-γ, Cat Number: ab46107, Abcam Company, Cambridge, MA, USA), immunoglobulin E (IgE, Cat Number: ab157736, Abcam Company, Cambridge, MA, USA), phospholipase A2 (PLA2, Cat Number: MBS262388, MyBioSource Inc., San Diego, California, USA) and total protein (TP, Cat Number: 128500, Pars Azmoon. Co., Iran) in the BALF were measured by enzyme-linked immunosorbent assay (ELISA) sandwich method with appropriate protocol recommended by company. The ratio of IFN-γ/IL4 as an index of Th_1_/Th_2_ was also calculated.

### Pathological evaluation

Histological examination was performed on right lung which was not lavaged. The right lung was fixed in 10% buffered formalin (37%, CAS Number: M103999.2500Merck, Germany). The preserved tissues were dehydrated, embedded in paraffin, sectioned at 3 μm thickness, and stained by hematoxylin-eosin (H&E) solution. Cut number per sample was 3 to 4 cut. The slides were examined under a light microscope (Model Nikon E200) by a blinded pathologist to the experimental groups for quantifying the extent of lung histopathological changes. The number of microscopic fields was 8–10 per section [28]. The pathological changes including interstitial inflammation, interstitial fibrosis, bleeding, plaque and emphysema were scored as 0: if there were not pathological changes, 1: if there was patchy changes and 2; when severe changes were present (in the most parts of the lung).

### Data analysis

Means ± SEM of the results were presented. Comparison between the results of control, sensitized and treated groups was done using one-way analysis of variance (ANOVA) with Tukey-Kramer’s post-test. For statistical analysis, InStat (GraphPad Software, Inc., La Jolla, USA) package was used. Statistical significance was considered if *P* values were less than 0.05.

## Results

### The effect of the *O. basilicum* extract on BALF levels of IFN-γ, IL-4 and IgE

**Fig. 2:**
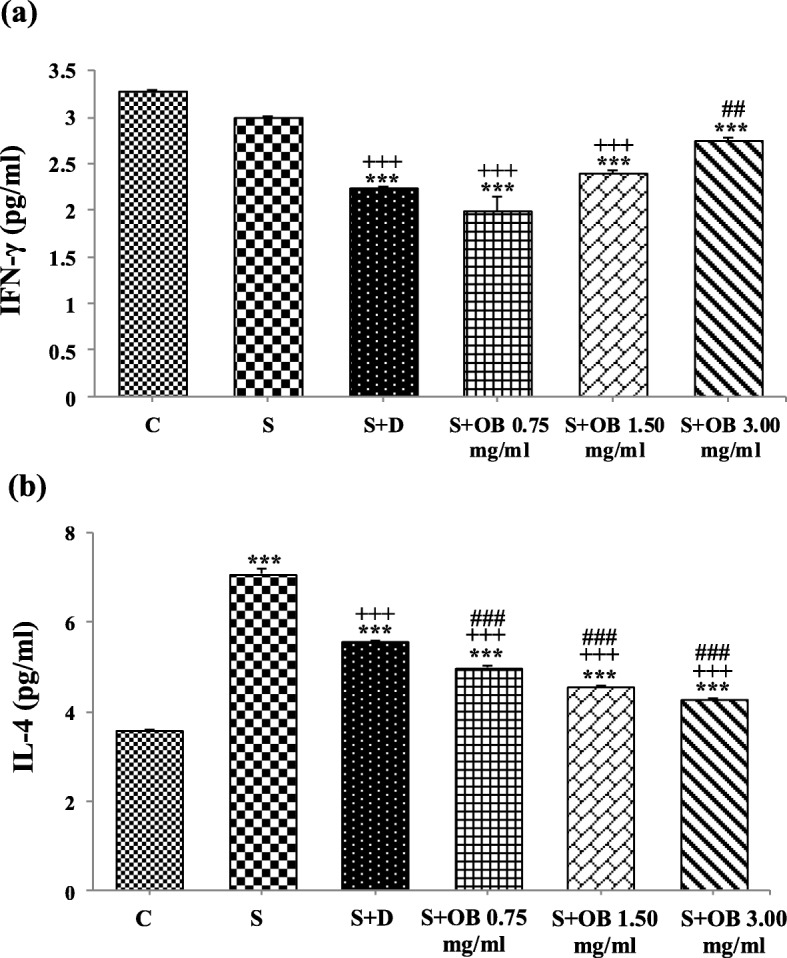
Bronchoalveolar lavage fluid levels of IFN-γ (**a**) and IL-4 (**b**), (mean ± SEM) in the control (C), sensitized (S), treated sensitized animals with dexamethasone (S + D) and three *O. basilicum* concentrations (S + OB) groups, (in each group, *n* = 8). ***: *P* < 0.001, comparison with C group. +++: *P* < 0.001, comparison with S group. ##: *P* < 0. 01, ###: *p* < 0.001, comparison with S + D group. One-way analysis of variance (ANOVA) with Tukey-Kramer’s post-test was used for statistical analysis

BALF level of IFN-γ in S group was not significantly different compared to C group. There were significant decrease in BALF level of IFN-γ in S + D, S + OB 0.75, S + OB 1.50 and S + OB 3.00 groups compared to C group (all, *P* < 0.001). Treatment of sensitized rats with two low concentrations of the extract and dexamethasone led to significant decrease in IFN-γ as compared to S group (*P* < 0.001 for all). A significant difference in BALF level of IFN-γ between S + OB 3.00 and S + D groups was also seen (*P* < 0.001), (Fig. 2a).

BALF level of IL-4 in S group was significantly higher than C group (*P* < 0.001). BALF level of IL-4 in S + D, S + OB 0.75, S + OB 1.50 and S + OB 3.00 groups were significantly lower than S group (all, *P* < 0.001). There reduction of IL-4 level in S + OB 0.75, S + OB 1.50 and S + OB 3.00 groups were significantly more than S + D group (all, *P* < 0.001), (Fig. 2b).

**Fig. 3:**
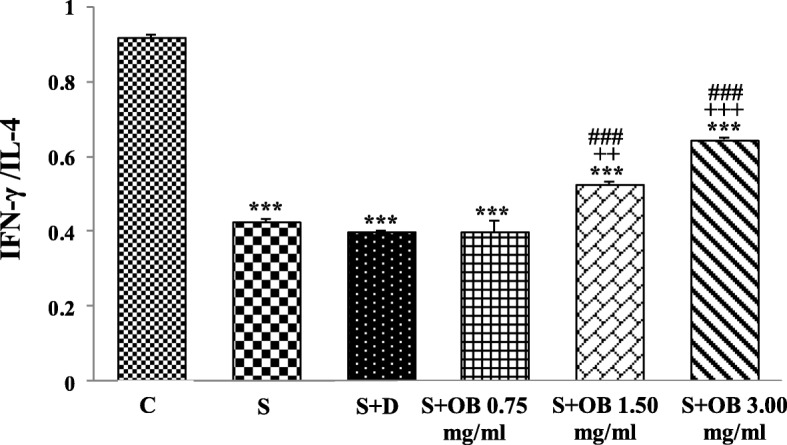
Values (mean ± SEM) of IFN-γ to IL-4 ratio in the control (C), sensitized (S), treated sensitized animals with dexamethasone (S + D) and three *O. basilicum* concentrations (S + OB) groups, (in each group, *n* = 8). ***: *P* < 0.001, comparison with C group. ++: *P* < 0.01, +++ *P* < 0.001, with S group. ###: *P* < 0.001, comparison with S + D group. One-way analysis of variance (ANOVA) with Tukey-Kramer’s post-test was used for statistical analysis

The ratio of IFN-γ/IL-4 was decreased in all untreated and treated sensitized groups compare to C group (all, *P* < 0.001). However, the ratio of IFN-γ/IL-4 in sensitized rats treated with 1.5 and 3.0 mg/mL of the extract was significantly increase compared to S group (*P* < 0.01 and *P* < 0.001, respectively). The ratio of IFN-γ/IL-4 in S + OB 1.50 and S + OB 3.00 groups was also significantly higher compared to S + D group (*P* < 0.001 for both), (Fig. 3).

**Fig. 4:**
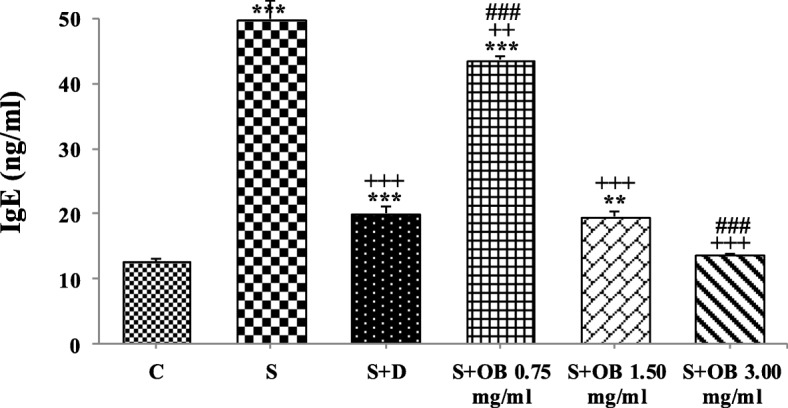
Bronchoalveolar lavage fluid level of IgE (mean ± SEM) in the control (C), sensitized (S), treated sensitized animals with dexamethasone (S + D) and three *O. basilicum* concentrations (S + OB) groups, (in each group, *n* = 8). **: *P* < 0.01, ***: *P* < 0.001, comparison with C group. ++: *P* < 0.01, +++: *P* < 0.001, comparison with S group. ###: *P* < 0.001, comparison with S + D group. One-way analysis of variance (ANOVA) with Tukey-Kramer’s post-test was used for statistical analysis

BALF IgE levels were significantly higher in S, S + D, S + OB 0.75 (all, *P* < 0.001), and S + OB 1.50 (*P* < 0.01) groups compared to that of C group. The BALF IgE levels in S + D, S + OB 0.75, S + OB 1.50 and S + OB 3.00 groups were significantly decreased compared to S group (*P* < 0.01 to *P* < 0.001). BALF IgE level in treated group with high concentration of the extract was also lower than S + D group (*P* < 0.001), (Fig. 4).

The effects of 1.5 and 3.0 mg/mL concentrations of the extract on all immunological mediator levels were significantly higher than the effect of its low (0.75 mg/mL) concentration (*P* < 0.05 to *P* < 0.001). The effect of 3.0 mg/mL of the extract on all immunological mediator levels was also significantly higher than the effect of 1.5 mg/mL of the extract (*P* < 0.05 to *P* < 0.001, Table 2).

**Table 2:** IFN-γ, IL-4, IFN-γ/IL-4 and IgE values in sensitized group treated with three different concentrations of *O. basilicum* (S + OB)

Groups	IFN-γ (pg/ml)	IL-4 (pg/ml)	IFN-γ/IL-4	IgE (ng/ml)
S + OB 0.75 mg/ml	1.99 ± 0.15	4.99 ± 0.04	0.40 ± 0.03	43.66 ± 0.56
S + OB 1.50 mg/ml	2.39 ± 0.03 ^+^	4.55 ± 0.03 ^+++^	0.53 ± 0.01 ^++^	19.49 ± 0.74 ^+++^
S + OB 3.00 mg/ml	2.75 ± 0.03 ^+++ #^	4.27 ± 0.04 ^+++ ##^	0.64 ± 0.01 ^+++ ##^	13.70 ± 0.11 ^+++ ###^

^+^: *p* < 0.05, ^++^: *p* < 0.01, ^+++^: *p* < 0.001, comparison with S + OB 0.75 group. ^#^: *p* < 0.05, ^##^: *p* < 0.01, ^###^: *p* < 0.001, comparison between S + OB 3.00 and S + OB 1.50 groups. Values are expressed as mean ± SEM. One-way analysis of variance (ANOVA) with Tukey-Kramer’s post-test was used for statistical comparisons, (in each group, *n* = 8)

### The effect of the *O. basilicum* extract on BALF levels of PLA2 and TP

**Fig. 5:**
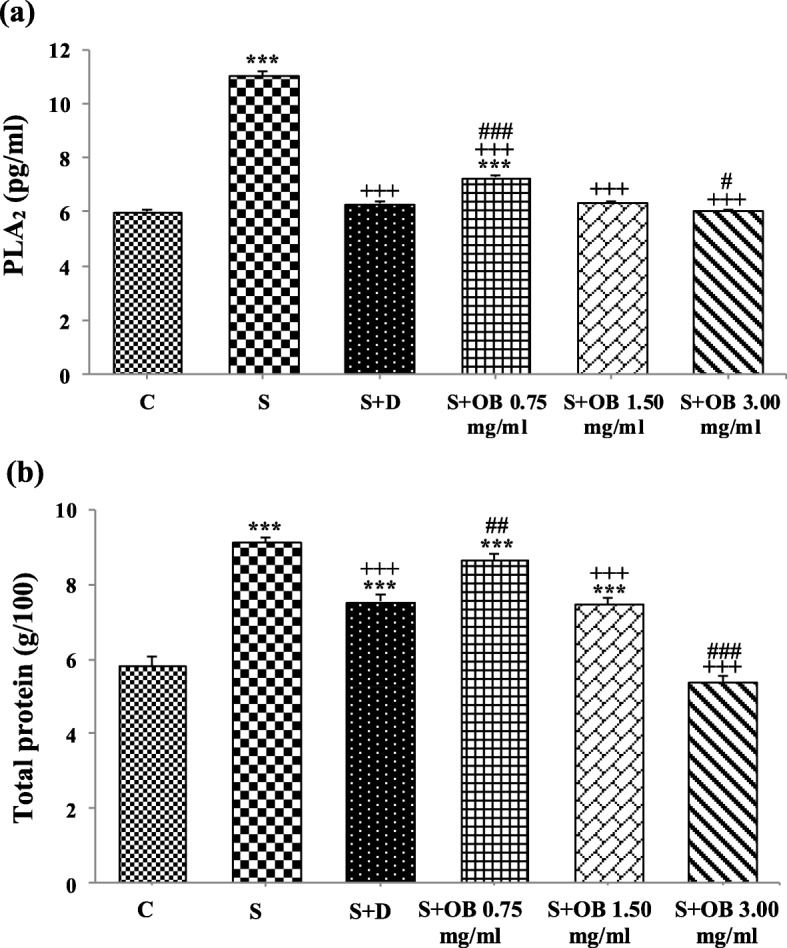
Bronchoalveolar lavage fluid levels of PLA_2_ (**a**) and total protein (**b**), (mean ± SEM) in the control (C), sensitized (S), treated sensitized animals with dexamethasone (S + D) and three *O. basilicum * concentrations (S + OB) groups, (in each group, *n* = 8). ***: *P* < 0.001, comparison with C group. +++: *P* < 0.001, comparison with S group. #: *P* < 0.05, ##: *P* < 0.01, ###: *P* < 0.001, comparison with S + D group. One-way analysis of variance (ANOVA) with Tukey-Kramer’s post-test was used for statistical analysis

BALF level of PLA_2_ in S group were significantly higher than C group (both, *P* < 0.001). The level of PLA2 was decreased in all treated groups (all, *P* < 0.001). PLA2 value in S + OB 0.75 was higher but in S + OB 3.00 groups showed significantly lower compared to S + D group (*P < 0.001* and *P* < 0.05, respectively), (Fig. 5a).

BALF level of TP in S group was significantly higher than C group (*P* < 0.001). The level of TP was decreased in S + D, S + OB 1.50 and S + OB 3.00 groups compare to S group (all, *P* < 0.001). The mean value of TP in S + OB 3.00 group was significantly lower compared to S + D group (*P < 0.001*). There was also no significant difference between S + OB 3.00 and C groups, (Fig. 5b).

The effects of 1.5 and 3.0 mg/mL of the extract on PLA_2_ and TP levels were significantly higher than the effect of its low (0.75 mg/mL) concentration (*P* < 0.01 to *P* < 0.001). The high extract concentration effect on PLA_2_ and TP levels was significantly higher than the medium concentration effect (*P* < 0.05 to *P* < 0.001, Table 3).

**Table 3:** PLA_2_ and total protein (TP) values in sensitized group treated with three different concentrations of *O. basilicum* (S + OB)

Groups	PLA_2_ (pg/ml)	TP (g/100)
S + OB 0.75 mg/ml	7.28 ± 0.078	8.67 ± 0.18
S + OB 1.50 mg/ml	6.35 ± 0.04 ^+++^	7.48 ± 0.17 ^++^
S + OB 3.00 mg/ml	6.06 ± 0.032 ^+++ #^	5.37 ± 0.16 ^+++ ###^

^++^: *p* < 0.01, ^+++^: *p* < 0.001, comparison with S + OB 0.75 group. ^#^: *p* < 0.05, ^###^: *p* < 0.001, comparison between S + OB 3.00 and S + OB 1.50 groups. Values are expressed as mean ± SEM. One-way analysis of variance (ANOVA) with Tukey-Kramer’s post-test was used for statistical comparisons, (in each group, *n* = 8)

### The effect of the *O. basilicum* extract on lung pathological changes

**Fig. 6:**
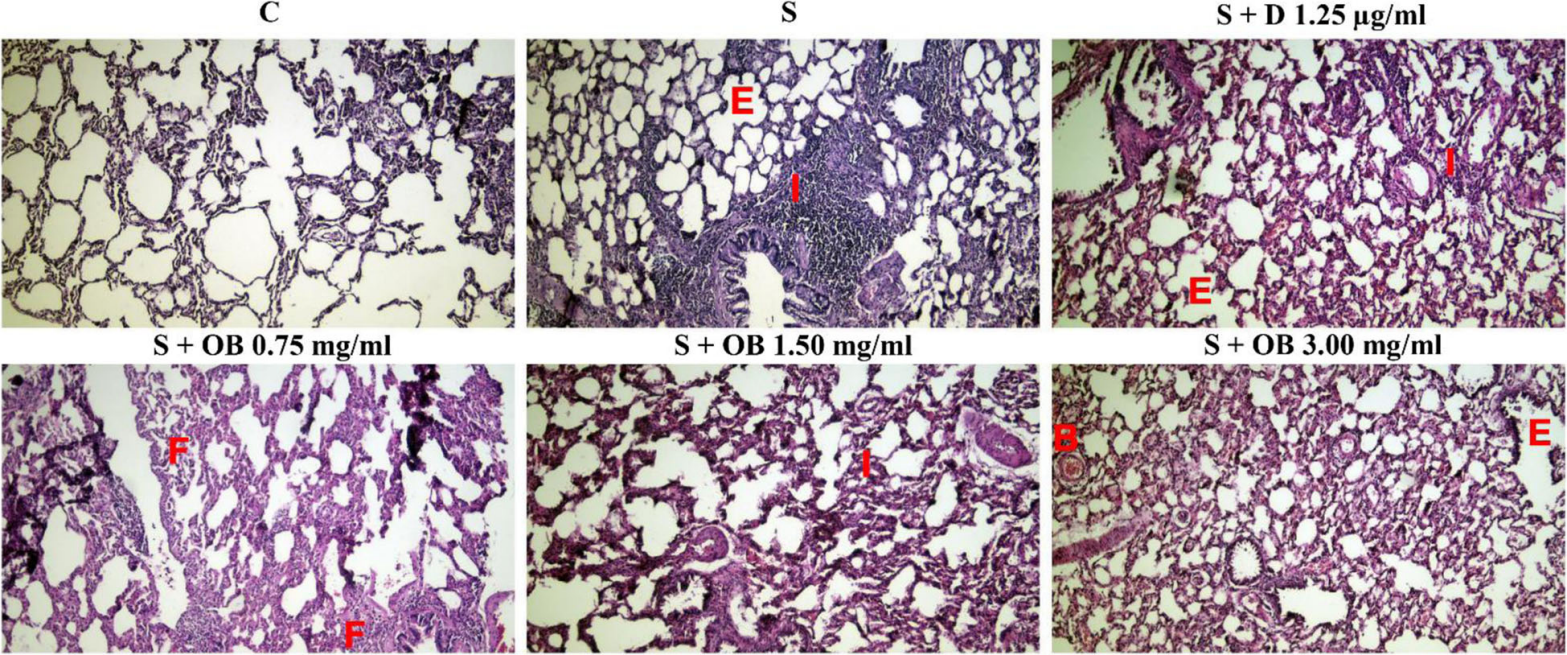
Photograph of a lung specimen with interstitial inflammation (I), interstitial fibrosis (F), bleeding (B) and emphysema (E) in the control (C), sensitized (S), treated sensitized animals with dexamethasone (S + D) and three *O. basilicum* concentrations (S + OB) groups, (magnification 100×)

**Fig. 7:**
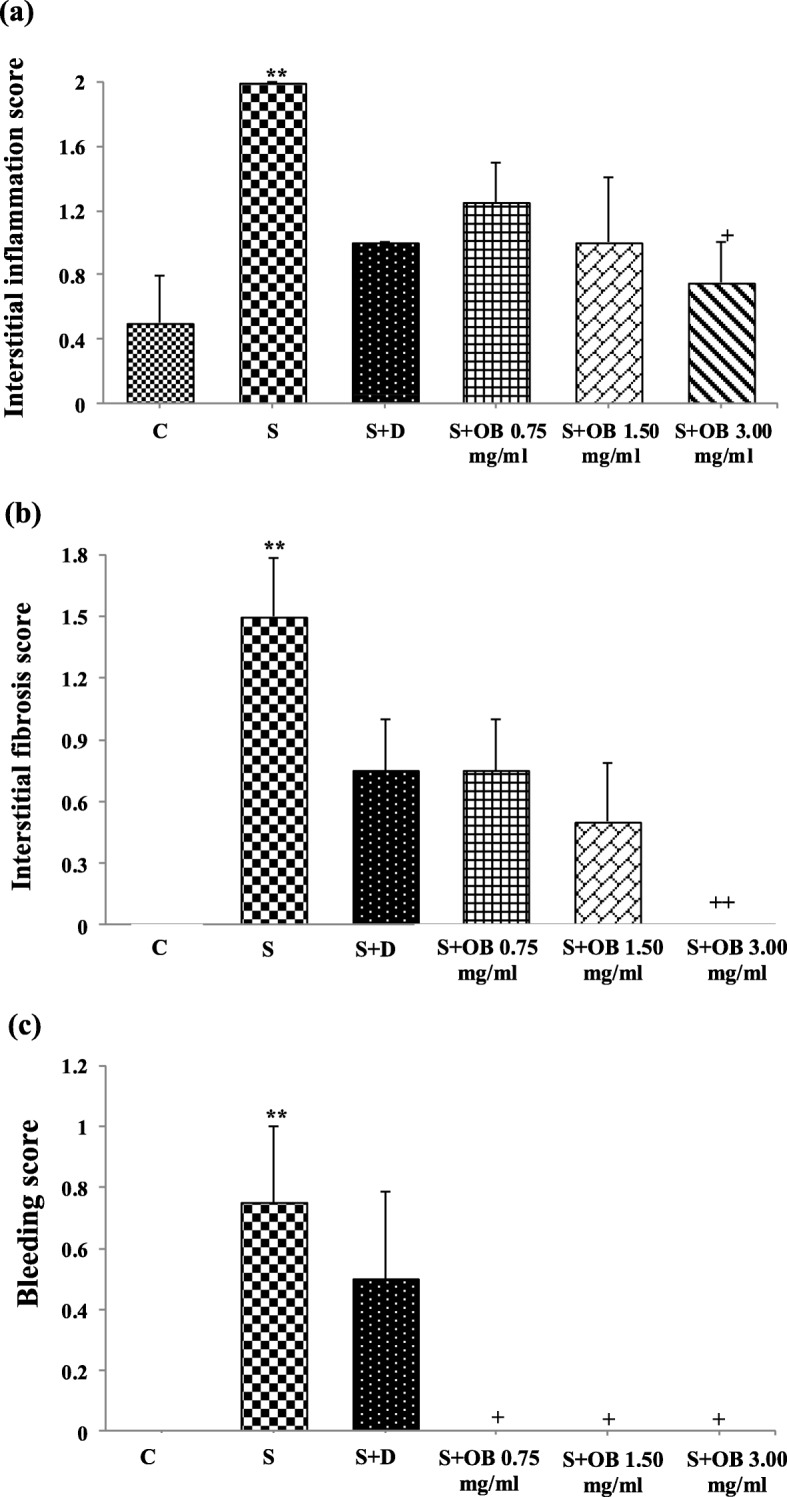
The score of interstitial inflammation (**a**), interstitial fibrosis (**b**) and bleeding (**c**) in the control (C), sensitized (S), treated sensitized animals with dexamethasone (S + D) and three *O. basilicum* concentrations (S + OB) groups, (in each group, *n* = 8). *: *P* < 0.05, **: *P* < 0.01, comparison with C group. +: *P* < 0. 05, ++: *P* < 0.01, comparison with S group. One-way analysis of variance (ANOVA) with Tukey-Kramer’s post-test was used for statistical analysis

**Fig. 8:**
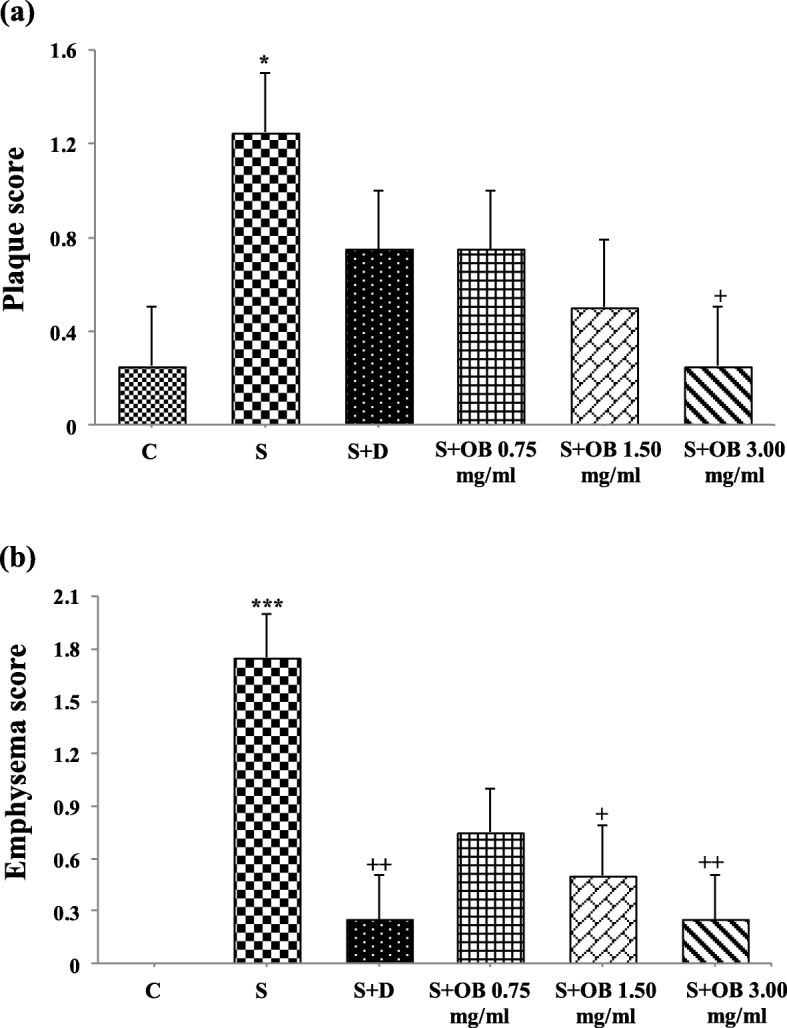
The score of airway plaque (**a**) and emphysema (**b**) in the control (C), sensitized (S), treated sensitized animals with dexamethasone (S + D) and three *O. basilicum* concentrations (S + OB) groups, (in each group, *n* = 8). *: *p* < 0.05, ***: *p* < 0.001, comparison with C group. +: *p* < 0. 05, ++: *p* < 0.01, comparison with S group. One-way analysis of variance (ANOVA) with Tukey-Kramer’s post-test was used for statistical analysis

All pathological scores in the S group, were significantly higher than C group (*P* < 0.05 to *P* < 0.001, Figs. 6, 7 and 8).

High extract concentration treatment caused significant improvement in interstitial inflammation, interstitial fibrosis and plaque but extract concentrations treatment caused significant improvement in bleeding and treatment with dexamethasone and two higher extract concentrations caused significant improvement in emphysema of sensitized rats (*P* < 0.05 to *P* < 0.01, Fig. 8). There was no significant difference in pathological changes between C group and treated groups with dexamethasone and all concentrations of the extract (Fig. 7).

The effects of treatment with three concentrations of the extract on all pathological changes have no significant difference with each other (Table 4). No significant difference was observed between dexamethasone treatment and all extract concentrations treatment on pathological changes (Figs. 7 and 8).

**Table 4:** Pathological changes in sensitized group treated with three different concentrations of *O. basilicum* (S + OB)

Groups	Interstitial inflammation	Interstitial fibrosis	Bleeding	Plaque	Emphysema
S + OB 0.75 mg/ml	1.25 ± 0.25	0.75 ± 0.25	0 ± 0	0.75 ± 0.25	0.75 ± 0.25
S + OB 1.50 mg/ml	1 ± 0.4	0. 5 ± 0.29	0 ± 0	0. 5 ± 0.29	0.5 ± 0.29
S + OB 3.00 mg/ml	0.75 ± 0.25	0 ± 0	0 ± 0	0.25 ± 0.25	0.25 ± 0.25

Values are presented as mean ± SEM. Pathological changes were not significantly different on among different extract concentrations. One-way analysis of variance (ANOVA) with Tukey-Kramer’s post-test was used for statistical comparisons, (in each group, *n* = 8)

## Discussion

Significant increases in IL-4, IgE, PLA_2_ and TP levels, significant decrease in IFN-γ/IL-4 ratio, increased all pathological changes were seen in a rat model of asthma compared to control group. These changes indicated the induction of a rat model of asthma which supported by previous studies [29–31]. There are three phases in allergic asthma development including 1) Induction phase in which T helper cytokines play a role in the development of asthma, 2) Early-phase asthmatic reaction (EAR) in which preformed mediators, newly synthesized lipid mediators and cytokines are the main mediators, and 3) Late-phase asthmatic reaction (LAR) in which neutrophils, eosinophils, T cells, macrophages, dendritic cells, and structural cells produce inflammatory molecules and lead to inflammation and structural changes in airway well [32]. The results of the current study indicated the induction of all three phases of development of asthma.

Airway inflammation is the main characteristic feature of asthma, and recent strategies for managing asthma have been emphasized on suppression of Th2-mediated airway inflammation [3]. Therefore, preventive treatments should minimize the airway inflammation caused by asthma.

Most of the above-mentioned variables were improved by administration of *O. basilicum* and dexamethasone. Anti-inflammatory effect of dexamethasone on respiratory tract in asthmatic mice [33] and anti-inflammatory activity of *O. basilicum* against different mediator-induced paw edema in rats through blocked of arachidonic acid metabolism, both cyclooxygenase and lipoxygenase pathways [18] has been shown, supporting the results of this study.

Various plant constituents including glycosidic structures, flavonoides and saponines showed anti-inflammatory activity [34]. In *O. basilicum* leaves, flavonoids such as quercetin, isoquercetrin, kaempferol and rutin, and glycosids like esculin and syringin have been found [35] that may be responsible for the anti-inflammatory activity. *O basilicum* is one of the most important aromatic plants with aromatic compounds including 4-allylphenol, anethole, anisaldehyde, benzyl alcohol, cuminaldehyde, ethyl cinnamate, methyl benzoate, methyl cinnamate, methyl eugenol, methyl salicylate, phenethyl alcohol, phenyl acetaldehyde, safrole, benzaldehyde and estragole [35]. Anti-inflammatory effects of the *O. basilicum* essential oil and its main compound estragole [36], and volatile constituents extracted from the leaves of *O. basilicum* such as eucalyptol, linalool, borneol acetate, alpha-bergamoten, germacrene and a triterpenoid alpha-amyrin were previously reported [37–39]. However, further studies are needed to demonstrate which one of these chemical compounds from *O. basilicum* are the most important chemicals for its anti-inflammatory effects.

An imbalance of Th1/Th2 cells can lead to tracheal hyper-responsiveness and inflammation. Asthmatic airway inflammation can be modulated by The Th1/Th2 balance [3]. Immunomodulatory properties of aqueous extract of this plant and some of its constituents on human immune cells suggest the potent natural immunomodulatory of these influencing several types of immune-responses and may have potential health benefits [21]. *O. basilicum* extract improved histological, morphometric and immunohistochemical changes induced by cadmium in albino rats [40]. Immuno-stimulant effects of this plant against gibberellic acid and auxin supplementation in broilers ration [41] were also supported the results of the present study. Therefore, the plant extract may have therapeutic effect on inflammatory diseases via reduction of involved cells in the respiratory tract inflammation.

This study also demonstrated that the effects of the plant extract on various variables in sensitized rats were concentration dependent. In fact, the effects of two higher concentrations of the extract on all immunological and inflammatory mediators levels were significantly higher than the effect of its low concentration and the effect of high extract concentration was significantly higher than its medium concentration.

In treated group with high extract concentration, its effect on the levels of IFN-γ, IgE, PLA_2_ and TP, in treated groups with all extract concentrations, their effects on IL-4 level and the in treated groups with two higher extract concentrations, their effects on the ratio of IFN-γ to IL-4 were significantly higher than dexamethasone treatment. However, the effects of low extract concentration on IgE, PLA_2_ and TP levels were significantly lower than dexamethasone treatment. These results showed comparable or even more potent preventive effect of the extract compared to the dexamethasone effect. These findings also indicated more specific effect of the extract on IFN-γ/IL-4 ratio and therefore on Th_1_/Th_2_ balance compared dexamethasone treatment in sensitized rats.

The effect of the extract on immunological changes including IFN-γ, IL-4, IgE, PLA2 and TP levels and the ratio of IFN-γ/IL4 as an index of Th1/Th2 as well its effect on pathological changes were shown in the current study. In previous studies, the effect of *O. basilicum* extract on depression like behavior, tracheal responsiveness, lung inflammatory cells and oxidant-antioxidant biomarkers in the OA-sensitized rats was shown [23, 25, 26] which support the findings of the present studies. However, this study has some limitations such as the assessment of qualitative and quantitative extract composition which should be examined in further studies.

## Conclusions

Anti-inflammatory and immunomodulatory effects of *O. basilicum* extract on asthma were shown by increasing the IFN-γ/IL-4 ratio (Th_1_/Th_2_ balance) and decreasing BALF levels of IgE, PLA_2_ and TP as well as improvement of pathological changes in sensitized rats by the plant. These findings suggest the preventive therapeutic potential for the *O. basilicum* extract on animal model of asthma.

## Data Availability

All the data supporting contained within the manuscript. Detailed data could be also obtained from corresponding author.

## Abbreviations

BALF: Bronchoalveolar lavage fluid, IFN-γ Interferon-γ
IgE: Immunoglobulin E
IL-4: Interleukin 4
LAR: Late-phase asthmatic reaction
OB: *Ocimum basilicum*
OA: Ovalbumin
PLA2: Phospholipase A2
TP: Total protein
